# Virmid: accurate detection of somatic mutations with sample impurity inference

**DOI:** 10.1186/gb-2013-14-8-r90

**Published:** 2013-08-29

**Authors:** Sangwoo Kim, Kyowon Jeong, Kunal Bhutani, Jeong Ho Lee, Anand Patel, Eric Scott, Hojung Nam, Hayan Lee, Joseph G Gleeson, Vineet Bafna

**Affiliations:** 1 Department of Computer Science and Engineering, University of California at San Diego, 9500 Gilman Drive, La Jolla, CA 92093, USA; 2 Department of Electrical and Computer Engineering, University of California at San Diego, 9500 Gilman Drive, La Jolla, CA 92093, USA; 3 Institute for Genomic Medicine, Rady Children's Hospital, University of California at San Diego, 9500 Gilman Drive, La Jolla, CA 92093, USA; 4 School of Information and Communications, Gwangju Institute of Science and Technology, 123 Cheomdangwagi-ro, Buk-gu, Gwangju, 500-712, Republic of Korea; 5 Department of Computer Science, Stony Brook University, 100 Nicolls Road, NY 11794, USA; 6 Graduate School of Medical Science and Engineering, KAIST, 291 Daehak-ro, Yuseong-gu, Daejeon 305-701, Republic of Korea

## Abstract

Detection of somatic variation using sequence from disease-control matched data sets is a critical first step. In many cases including cancer, however, it is hard to isolate pure disease tissue, and the impurity hinders accurate mutation analysis by disrupting overall allele frequencies. Here, we propose a new method, Virmid, that explicitly determines the level of impurity in the sample, and uses it for improved detection of somatic variation. Extensive tests on simulated and real sequencing data from breast cancer and hemimegalencephaly demonstrate the power of our model. A software implementation of our method is available at http://sourceforge.net/projects/virmid/.

## Background

Identifying mutations relevant to a specific phenotype is one of the primary goals in sequence analysis. With the advent of massively parallel sequencing technologies, we can produce an immense amount of genomic information to estimate the landscape of sequence variations. However, the error rates for base-call and read alignment still remain much higher than the empirical frequencies of single nucleotide variations (SNVs) and *de novo *mutations [1]. Many statistical methods have been proposed to strengthen mutation discovery in the presence of confounding errors [2-4].

Finding somatic mutations is one particular type of variant calling, which constitutes an essential step of clinical genotyping. Unlike the procedures used for germ line mutation discovery, the availability of a matched control sample is indispensable. Here, sequence variants that exist in the control sample are used as a basis for measuring individual polymorphisms, while the disease-only mutations are generally regarded as candidate somatic mutations. Traditional approaches call variants from each sample to estimate the sequential differences [5,6]. But most recent studies that calculated joint probabilities of the disease-control genotype pairs had a higher efficiency in separating true somatic mutations from germ line mutations by considering correlations between two samples [7-9]. With the aid of probabilistic variant calling models, whole genome/exome sequencing data have been used to identify potential *de novo *mutations in various studies including schizophrenia [10], autism [11], and cancer [12].

However, there are many cases where mutation discovery might be confounded. One big hurdle is the impurity and heterogeneity of the disease sample. For example, gastric and breast cancer tissues usually contain large numbers of stromal cells, which makes the acquisition of a pure cancer sample infeasible [13]. More importantly, there are many cases in which this type of contamination is not only inevitable but dominates the sample constitution. Focal malformation of cortical developments, including focal cortical dysplasia and hemimegalencephaly, is the most common cause of childhood intractable epilepsy and there are disease cells in affected brain regions along with a high proportion of normal cells [14]. A similar problem arises when trying to detect a small amount of target genome mixed in control samples. In organ transplantation, an increased level of circulating cell-free DNAs (approximately 10%) from the donor in the recipient's blood indicates a higher risk of failure [15]. Cell-free DNAs are also found in pregnancy; a small number of fetal DNAs (approximately 13%) are detectable in maternal plasma [16]. In both cases, accurate identification of the target genotypes will provide a basis for a non-invasive and low-cost diagnostic method.

Conventional methods for somatic mutation profiling are severely compromised in highly contaminated samples because the abundant short reads originating from control genomes obscure the true allele frequency (AF) at the site of *de novo *mutations. This usually results in a failure to call the true variants. Two questions arise: (1) how can we estimate the contamination level, defined as the proportion of the control sample in the mixed disease sample? (α, 0 ≤ α ≤ 1) and (2) what is the effective way to use α in SNV calling. A natural approach to estimating α has been adopted by many previous studies [15,17-20]. For any heterozygous mutation, the B allele frequency (BAF) is expected to be close to 50%. A significant and consistent deviation from this value is indicative of the existence and level of control sample inclusion. We found, however, there are two substantial problems to this approach. First, it needs an initial SNV calling procedure either from sequencing or SNP array data, which takes extra time and cost. Second, and more importantly, the initial mutation call is not representative; a higher BAF is likely to be observed in the selected sites causing an underestimation of α. We will show that the bias is significantly large in highly contaminated samples and describe a way to resolve it. Incorporating the estimated α in the SNV calling model is another important problem. There are only a few studies that consider α or a similar concept in SNV calling [7,9]. More rigorous and explicit use of α by a tight parameterization in a probabilistic model will improve the accu-racy of final calls.

**Figure 1:**
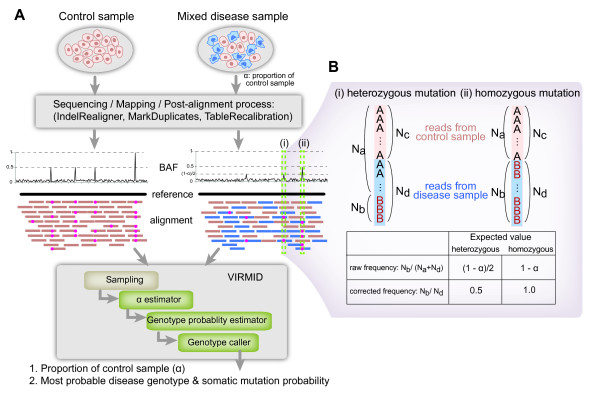
**Overall Virmid workflow**. **(a) **Disease/control paired data are used (top) to generate an alignment (BAM) file. The mixed disease sample produces short reads of mixed types (blue and orange rectangles). Somatic mutations, where the control has the reference genotype (AA) and the disease has the non-reference (AB or BB, red dots in the alignment), are hard to detect if there is high contamination due to the significant drop in B allele frequency (BAF). Virmid takes the disease/control paired data and analyzes: (1) the proportion of control cells in the disease sample (α) and (2) the most probable disease genotype for each position that can be used to call somatic mutations. **(b) **An example BAF drop. Without contamination, the expected BAF is 0.5 and 1.0 for heterozygous and homozygous mutations sites, respectively. When there is control sample contamination, α, mutation alleles are observed only in (1 - α) of the whole reads. So the expected BAF drops to (1 - α)/2 and (1 - α). With an accurate estimate of α, Virmid can detect more true somatic mutations, which would be missed by conventional tools due to insufficient observation of B alleles. BAF, B allele frequency.

Here we describe a novel probabilistic model Virmid (virtual microdissection for variant calling) which estimates: (1) the sample contamination level (α) and (2) the disease genotypes including somatic mutations (Figure 1). At the core of Virmid lies a maximum likelihood estimator (MLE) of α and the joint probabilities of control and disease genotypes represented by joint genotype probability matrix G (see Materials and methods), driven from a local distribution of BAF. It does not require any prior SNV calling nor does it attempt to find variants beforehand; we show that this not only saves computation but also greatly improves the accuracy by reducing sampling bias. Our model also accounts for other sources of noise including sequencing error, mapping error, read mapping bias and mappability [21] of the genomic regions for more accurate modeling, as well as the effect of copy number variations. More importantly, the tight coupling of α and G implemented in a single integrated model enables rigorous recalibration of genotype probabilities with the given α. We demonstrate using simulated and real exome sequencing data that Virmid significantly increases overall precision and recall in variant finding. Even in some intractable cases, where the target genome exists in a very small amount (α ≥ 80%) in the sample, Virmid shows a near-robust performance. We expect that this improvement will be useful for many related problems from cancer somatic mutation profiling to contaminant genome identification.

## Results and discussion

The Virmid workflow is shown in Figure 1. The input to Virmid includes short reads sequenced from a pure control sample and a potentially mixed disease sample. As a preprocessing step, the reads are aligned to the reference genome to generate sequence alignments. Second, the alignments are corrected using post-processing tools such as the Genome Analysis Toolkit's (GATK) IndelRealigner [22]. Third, BAF is calculated from the corrected alignments for every nucleotide position. Fourth, initial filters are applied for quality control as well as to reduce sample size. Due to the large size of the usual genomic data, we implemented a multi-tier sampling strategy (see Figure S1 in Additional file 1), which reduces the overall running time and disk usage about seven to tenfold. Finally, two filtered alignment files (in pileup format) from the control and disease samples are prepared as input.

The first step for Virmid is to estimate α. Here we use A for the reference allele and B for the non-reference. The set of diploid genotypes is, thus, given by *G *= {AA, AB, BB}. As α is a global parameter, which affects all positions equally, a small subset of positions is sufficient for the estimation. To obtain robust and unbiased estimates, we use a number of criteria: (1) we use only the positions with no B allele in the controls to maximize the chance of getting true somatic mutations; (2) we eliminate positions with a very high or low coverage suggestive of a copy number variation (CNV); (3) more filters are applied so that the selected positions have mapping and sequencing quality values above a certain threshold and (4) the known mappability [21] of the corresponding reference region has to be above a certain threshold (see Materials and methods for detailed settings in Virmid). Finally, the sites are filtered to remove alleles with BAF lower than a parameter *R *(0 <*R *≤ 1). While this makes the filtered list biased for higher BAF mutations, the explicit parameter value *R *is incorporated in our model to correct that bias (see Materials and methods).

Virmid estimates α from the sampled sites using an MLE [23] with a gradient descent search and simultaneously estimates the joint genotype probability matrix G, based on the estimated α. The estimated α value and the matrix G are used to call the most likely genotype at every nucleotide position. Finally, somatic mutation filters are applied to reduce the number of false positives (see Materials and methods). The overall pipeline including data preprocessing is implemented as a single Java program. We utilized open source libraries such as SAMtools [2] and Picard [24] to increase the efficiency and compatibility of the program. We also significantly reduced the amount of memory and disk usage by minimizing the generation of temporary files.

### Test on simulated data

Simulated control and disease genomes were prepared from human chromosome 1 (hg19) by introducing random mutations. Out of 275,814 germ line (mutation rate: 10^-3^) and 2,522 somatic mutations (mutation rate: 10^-5^), 47,796 and 257 mutations were located in non-detectable regions (for example, because the reference genotype was unavailable) leaving only 228,018 and 2,265 mutations as a true answer set. Disease samples were generated by artificially mixing two genomes in 11 different proportions (α = 1%, 5%, 10%, 20%, 30%, 40%, 50%, 60%, 70%, 80% and 90%). The Illumina-like short reads (read length = 100 bp) in a medium (40×) coverage were mapped to the reference and used as the input in the Virmid pipeline (see Materials and methods for the complete protocol).

#### Contamination level estimation

**Figure 2:**
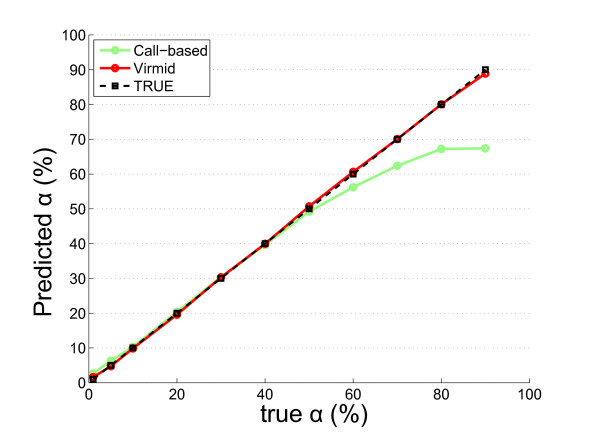
**Estimation of contamination**. Estimation of contamination level in a mixed disease sample. The propor-tion for the control sample (α) is estimated from the simulated mixed data. A total of 11 data sets with different α values (1%, 5%, 10% 20%, 30%, 40%, 50%, 60%, 70%, 80% and 90%) were generated and tested. Virmid estimated all the α values (red line with circles) with high concordance compared to the true values (black line with squares). Note that there is a significant bias in highly contaminated samples (α ≥ 60) in the call-based method (green line with circles) due to undetectable low BAF mutations; somatic mutations with higher BAF are likely to be called initially causing overestimation of BAF and underestimation of α.

The estimated sample contamination levels for the 11 different mixtures are shown in Figure 2 (red line with circles) and Table 1. The overall accuracy was near perfect with only 0.53% mean deviation from the true value. To test robustness, we ran Virmid 20 times for each data set varying the sampling parameter *R *(minimum BAF to control the number of sampling points). All replicated results were bounded within 2% (0.19 ≤ standard deviation ≤ 0.53), showing that the estimation with MLE is robust (Table 1). We found there is only a minor (± ~1) overestimation in very lowly (α ≤ 5%) and underestimation in very highly (α ≥ 80%) contaminated samples. However, the error size was negligible compared to a conventional call-based calculation (see Materials and methods), which estimates α based on initially identified somatic mutations (Figure 2, green line with circles).

**Table 1:** Accuracy and robustness of estimated α.

True α (%)	Estimated α (%)			Standard deviation (10^-2^)	α range	BAF range^b^
				
	Call based	**Virmid (/LOR)**^**a**^	Virmid			
1	2.64	2.56	1.61	0.19	1.23-1.90	11.11-47.50
5	6.31	5.56	4.74	0.28	4.07-5.17	11.11-46.34
10	10.3	10.50	9.86	0.51	9.34-11.03	11.43-44.12
20	20.4	19.92	19.59	0.44	18.44-20.13	11.43-38.46
30	30.4	30.33	30.28	0.48	28.48-30.79	11.11-33.33
40	39.6	40.46	39.94	0.22	39.49-40.51	11.43-28.89
50	49.2	51.38	50.72	0.23	50.46-51.31	11.11-23.53
60	56.2	61.16	60.62	0.54	59.78-61.48	10.71-19.15
70	62.4	70.28	70.05	0.16	69.82-70.44	10.00-14.29
80	67.2	80.33	80.04	0.38	79.52-80.76	9.38-10.81
90	67.4	88.91	88.88	0.53	88.06-90.00	8.82-9.68

^a^Estimates without the loss of read (LOR) bias correction. The probability that a read with at least one A is mappable was set to equal the probability that a read with at least one B is mappable, that is, *x*(A) = *x*(B) (see Material and methods). Note that loss of variants (LOV) bias has already been addressed.

^b^Range of minimum BAF in the observed data. The more data points Virmid observes, the smaller the minimum BAF obtained, and this is used in Virmid to correct sampling bias.

We note two types of biases in the call-based method (see Figure 2, green line), loss of reads (LOR) and loss or variants (LOV), which lead to overestimation and underestimation of α, respectively. LOR originates from the difference of mappability among short reads at the site of somatic mutation. Assume a disease genome has a heterozygous somatic mutation (AB) at position *i*. As the reference genome has an A genotype, reads with A at position *i *are more likely to be mapped. This results in under-representation of the B allele, followed by an overestimation of α (see Materials and methods for details). LOV is caused by the tendency that variant calling is more favorable in regions of higher BAF. Assume that a disease sample of α contamination has AB heterozygous mutations. In these positions, BAF follows a binomial (or similar) distribution with a probability of choosing the B allele of (1 - α)/2. In conventional SNV calling algorithms, the positions with higher BAF are easier to discover. Therefore, the distribution of BAF of the called mutations is shifted upward, which results in over-representation of the B allele, followed by underestimation of α.

The effects of the two estimation biases are dependent on α. The difference in the number of mapped reads with the A and B alleles is proportional to the absolute number of reads generated from the disease genome. So, the LOR bias is inversely proportional to α. On the other hand, the LOV effect is proportional to α because the SNV calling performance remains robust in the low contamination samples. The combined effect explains the bimodal error distribution of the call-based method. Eventually, the estimation result showed the suggested biases exist and are corrected efficiently in Virmid (see Materials and methods).

**Figure 3:**
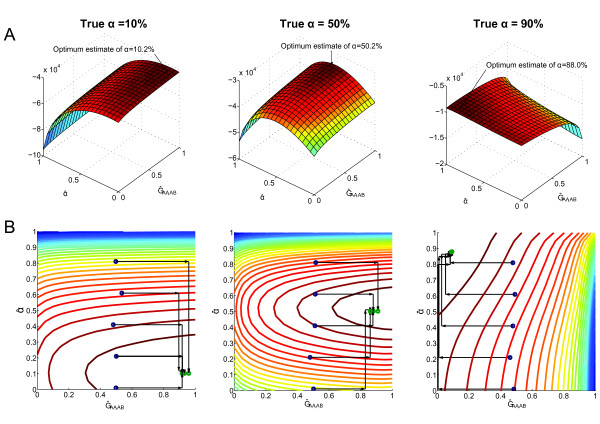
**Maximum likelihood estimation and search**. **(A) **Log likelihoods over different values of $\hat{\alpha }$ (the es-timate of α) and  Ĝ_AA,AB _(the estimate of G_AA,BB_, the probability that the control genotype is AA and the disease genotype is AB). The surface graph shows that likelihoods are maximized around the true α values (10% (left), 50% (middle), 90% (right)). **(B) **Search paths using the feasible direction method on contour maps. The method efficiently finds the optimum points (green circles) in only a few search steps. Searches from different starting points (blue circles) finally converge.

Because we do not rely on initial mutation calling, the sites used for α estimation may contain non-mutated positions. As we already filtered out all the positions where the control sample has one or more B alleles, only three possible joint genotypes remain: (AA-AA, AA-AB and AA-BB). Thus, Virmid estimates the frequencies of these genotypes along with α. Since the likelihood we used is dependent on α and the frequencies of the genotypes, we attempt to find the combination of α and the genotype frequencies that maximizes the likelihood. We showed empirically that the likelihood space is convex and maximized near the true answers (see Figure 3A and Figures S3 and S4 in Additional file 1 for all α values). Therefore, we used a fast gradient descent search algorithm to get MLE estimates, instead of slower Expectation-Maximization (EM) like algorithms (Figure 3B, see Materials and methods).

#### Somatic mutation calling

**Figure 4:**
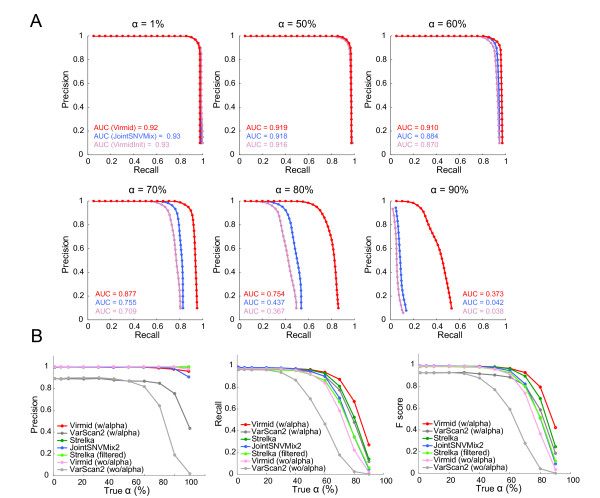
**Performance of somatic mutation detection**. Performance comparison of different methods for somatic mutation detection. **(A) **Precision-recall curves for Virmid with α (red), Virmid without α (light red) and JointSNVMix2 (blue) for six different α values (1%, 50%, 60%, 70%, 80% and 90%). Note that the performance is significantly improved when α is incorporated into the calling model. There is little difference in performance at low contamination levels (α ≤ 50). **(B) **Precision and recall scores of the final call generated for each α where mutation probabilities are not available; note that a single point instead of a curve is plotted for each α. As α increases, there is a consistent drop in precision, recall and F-score. The latter is given by: $\text{F}-\text{score}=2\times \frac{\text{precision}\times \text{recall}}{\text{precision}+\text{recall}}$ Four tools including Virmid, Strelka, VarScan2 and JointSNVMix2 were tested with the same data. Virmid and VarScan2 were tested in two different modes (with and without α). Strelka was also tested in two modes with or without applying quality control. Overall, Virmid with α had the best F-score, followed by Strelka, VarScan2 with α and JointSNVMix2. Note that the tools with α (Virmid with α, Strelka and VarScan2 with α) outperformed those without α (Virmid without α and VarScan2 without α), showing the importance of incorporating α in SNV calling. SNV, single nucleotide variation.

We ran Virmid to predict the most probable genotype for each nucleotide position in the simulated data set using the estimated α. Somatic mutations were called based on the predicted genotype probabilities after filtering. To evaluate the influence of α, we compared the result with those from other SNV calling tools including JointSNVMix2, Strelka and VarScan2 (Figure 4). Virmid and VarScan2 can take tumor purity and run in two different modes (with and without α); note that VarScan2 does not estimate α and so it was provided with our estimate. Strelka generates two outputs, a standard list and a filtered mutation list (see Materials and methods for a detailed protocol). Evaluation was done against the 2,265 true somatic mutations cataloged from a simulation procedure based on precision-recall curves (Figure 4A) where exact genotype probabilities are available (Virmid and JointSNVMix2), or a single precision, recall and F-score (Figure 4B) where only final mutation lists are provided (Strelka and VarScan2).

The performance of all algorithms was comparable for relatively low contamination (α ≤ 50), but varied considerably for higher α values. Generally, tools that incorporated the contamination level (Virmid with α, VarScan2 with α and Strelka, which has a non-explicit noise level that may indicate tumor purity) outperformed the ones that did not (Virmid without α, VarScan2 without α and JointSNVMix2). This is clearer when runs of the same tool with different values of α were compared (Virmid and VarScan2 with and without α).

**Figure 5:**
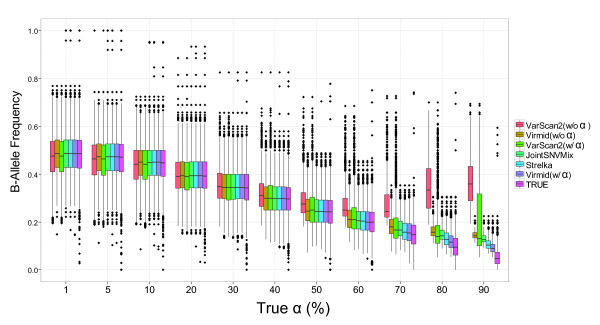
**BAF distribution of call sets**. BAF distribution of different call sets. Box plots are drawn for BAF in true (pink boxes) and called somatic mutations. From low to high contamination, the mean BAF decreases from 50% to 5%. Due to the difficulty in finding low BAF mutations, the call sets show a slight to significant increase in BAF. Virmid with α calculates BAF closest to that of the true set. Due to the undetectable true somatic mutations that contain no B alleles, there can be a large gap between true and call set BAF distributions (α = 80% and 90%). BAF, B allele frequency.

A detailed analysis of the BAF distribution in different call sets provides a second test of performance (Figure 5). Note that the mean BAF is given by (1 - α)/2. As expected, the BAF distribution of the true mutation set (Figure 5, pink bar) decreases as α increases; with low α, there is no major problem in detecting somatic mutations because BAF is high enough to be distinguished from non-mutational sequencing and mapping error frequencies. However, for high α, the algorithms start to fail in calling somatic mutations with relatively low BAF. The most extreme case is when no B allele is observed in the disease sample due to the low proportion of the true disease genome and its variance. For example, 316 out of 2,265 somatic mutation sites had no reads with the B allele in the sample with α = 90%. As there is no feasible way to detect these sites, the called mutation set must have a higher BAF distribution.

Finally, we revisit the coverage issue in SNV calling. Although moderate (40×) coverage is generally considered sufficient for SNV calling (calculated from [25]), high contamination needs higher coverage. For example, with 90% contamination, only 5% of reads (or two reads in 40× coverage) will sample the B allele. Higher coverage adds more confidence to each position's genotype probability by providing more reads to observe. To see the effect of higher coverage, we generated 100× simulation data sets from three highly contaminated data sets (α = 70%, 80% and 90%). The data sets were analyzed using Virmid. Table 2 shows the improvement in prediction performance (especially for recall). With 80% contamination, Virmid could identify 94% of the true somatic mutations with almost perfect accuracy. Even with 90% contamination, 68% of the true somatic mutations were discovered, which is more than 250% (611 to 1545) better than the 40 × coverage result with better precision (0.96 to 0.98). From the result, we can conclude that deeper coverage greatly improves the finding of mutations in highly contaminated samples and should be considered when sample purity is questionable.

**Table 2:** Improved mutation calling in higher coverage.

α^a^	n.Answer^b^	Coverage = 40 ×					Coverage = 100 ×				
		
		n.Predict^c^	n.Correct^d^	Precision	Recall	F-score	n.Predict^c^	n.Correct^d^	Precision	Recall	F-score
70%		1999	1976	0.98	0.87	0.93	2208	2198	1.00^e^	0.97	0.98
80%	2265	1551	1516	0.98	0.67	0.79	2142	2133	1.00^f^	0.94	0.97
90%		638	611	0.96	0.27	0.42	1572	1545	0.98	0.68	0.81

Precision and recall values are compared between two different coverages (40 × and 100 ×) at highly contaminated data (α = 70%, 80%, and 90%). Much deeper coverage is required when severe contamination is expected.

^a^α is the proportion of the control sample in the disease sample (contamination level).

^b^n.Answer is the number of true somatic mutations.

^c^n.Predict is the number of mutations predicted by Virmid.

^d^n.Correct is the number of correct mutations in the prediction.

^e^Rounded up from 0.9954.

^f^Rounded up from 0.9958.

Although testing on simulated data has a significant benefit through knowing the exact precision and recall of the true answer set, it has limitations. Many difficulties in somatic mutation detection arise from ambiguous read mapping. In simulation, the same reference genome assembly is used in artificial read generation. However, in real data, the donor genome contains significantly more variations other than SNVs, such as copy number variations and structural variations [26]. Therefore, we tested using publicly available disease data to give a more extensive validation of Virmid's performance.

### Tests on breast cancer data

To test with real disease data, we ran Virmid on 15 whole exome breast cancer data sets generated from The Cancer Genome Atlas (TCGA) project [27] (see Table S2 in Additional file 1 for the sample list). Breast cancer is known to contain a large number of stromal cells in the tumor mass [28], which makes the relevant genetic studies more challenging. In this context, estimating and considering the level of impurity might be necessary for a more accurate analysis of finding somatic mutations.

Before reporting the accuracy, the exact meaning of the sensitivity and specificity of a test must be defined. Note that in the absence of a complete list of true positives, predicted but unconfirmed calls cannot be treated as false positives. To test specificity, we generated false tumor/normal pairs from the same sample, where every call is a false positive. We applied a virtual tumor approach, suggested by the MuTect study [29] for the specificity test.

**Figure 6:**
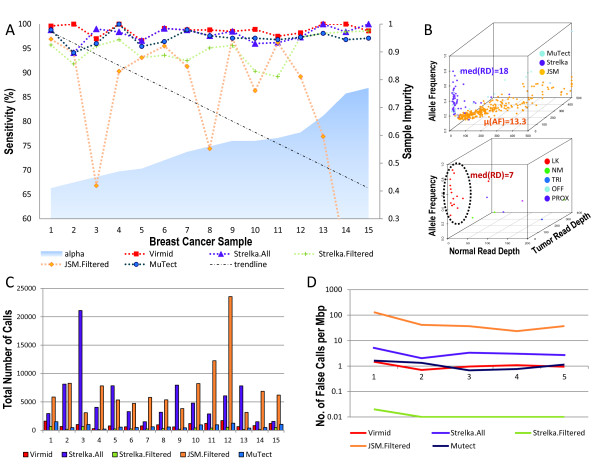
**Tests on 15 breast cancer exome sequencing data sets**. **(A) **Sensitivity results from four different tools (five different modes). The samples are ordered by our estimated α. **(B, upper) **Negative calls from Strelka (purple), MuTect (light blue) and JointSNVMix (orange). Each false negative resulted from a low allele frequency or low read depth. **(B, lower) **Many of the false negatives from Virmid (15/23) resulted from extremely low coverage in the normal sample, and there was insufficient likelihood of calling the somatic mutation (LK, red dots). The remaining eight false negatives were explained as filtering errors (see Materials and methods for more details of filtering). **(C) **Total number of calls. **(D) **Specificity result from the virtual tumor analysis. The number of false positive calls were normalized by the total size of the exome region to give the number of false positives per million base pairs. Note that the y-axis has a log scale. LK, likelihood in calling the somatic mutation; Mbp, million base pairs; NM, number of mismatches; OFF, read offset; PROX, proximity to an indel; TRI, tri-allele.

We first measured sample impurity by estimating α (Figure 6A, blue area). The values ranged from 0.41 to 0.77. Unlike for other monoclonal diseases, we note that there is a chance of overestimation due to the genetic heterogeneity in cancer (independently addressed in another study [19]). However, we found that the impurity range is generally consistent with the previous measurement from 21 breast cancers [28] and with TCGA's sample quality control step (see Materials and methods).

There were 1,654 experimentally validated mutations in the 15 exome data set (see Materials and Methods). Figure 6A shows the measured sensitivity of four different callers (Virmid, Strelka, JointSNVMix and MuTect); we excluded VarScan2 because it was used to generate the initial TCGA call set. The recently developed JointSNVMix has its own filtering module. We found the filtered version of JointSNVMix is always better than the unfiltered version because it does not miss a single true answer. So, we only used the filtered version of JointSNVMix in the comparisons. We found that the default Strelka filter eliminates most of the true answers (see Materials and methods) because it is too strict for exome sequencing data. So, we manually disabled its read depth filter. Out of 1,654 reads, Virmid only missed 23 validated mutations (36 for unfiltered Strelka, 47 for MuTect, 95 for filtered Strelka and 255 for JointSNVMix) and had the best sensitivity of 98.61% (97.82% for unfiltered Strelka, 97.16% for MuTect, 94.26% for filtered Strelka and 84.58% for JointSNVMix). As the pre-calculated α increases, a clear decrease of sensitivity was observed in JointSNVMix, which does not estimate sample impurity (Figure 6A, black trend line). The relatively smaller sensitivity increase of Virmid compared to Strelka and MuTect, which were shown to be consistent in the given α range, can be explained by other features such as unbiased estimation of sample purity and more rigorous filtering.

We then analyzed the types of negative calls. Out of 47, 95 and 255 false negative calls from filtered Strelka, MuTect and JointSNVMix, Virmid recovered 32, 81 and 252 mutations, which correspond to 68.1%, 85.3%, and 98.8% of the false negatives. The read depths (of the normal and tumor samples) and allele frequencies of the recovered mutations are shown in Figure 6B. We found the main reason for JointSNVMix missing calls was low allele frequency (orange dots in Figure 6B, upper, *μ *= 13.3%), while many of the missing calls for Strelka and MuTect resulted from low read depth (light blue and purple dots, median read depth = 18). Because the subtle changes in genotype probability are more critical at lower read depth, this results also proves the reliability of Virmid's genotype calculation model. We also analyzed the 23 Virmid's false negatives (Figure 6B, lower). In most cases (15/23), the read depth for the normal sample was extremely low (red dots in Figure 6B, lower, median read depth = 7). We found Virmid called these mutations as germ line mutations (AB-AB). Although they were missed as a consequence, we are convinced that our call is not theoretically wrong because at low read depth, the probability of finding only reference sequences from the AB genotype (solely by chance as calculated from a binomial distribution) is much higher than the prior probability of finding a somatic mutation (AA-AB). The only solution is to increase the read depth of the normal sample (for example, by balancing out), because calling these regions will greatly increase the false positive rate. Overall, the false negatives from Virmid were partially recovered by other tools (8, 9 and 20 by Strelka, MuTect and JointSNVMix, respectively).

In this test, sensitivity increases monotonically according to the total number of calls. It is informative to compare the number of calls needed to achieve a similar level of sensitivity. Figure 6C shows the total number of predicted mutations. We found that unfiltered Strelka (approximately 5,646 per sample) and JointSNVMix (approximately 7,362 per sample) predicted far more mutations than other systems (941, 336 and 738 per sample for Virmid, filtered Strelka and MuTect, respectively). Although we do not know if the non-validated calls were all false, we suspect there were more false positive calls made by those two tools.

To measure the specificity, we designed a virtual tumor by dividing high depth (> 80) normal samples into two artificial samples including one faked tumor and one faked control sample. Because all the reads were originally generated from the same genome, any positive calls on these samples can be considered as false positives. We ran all tools on five virtual tumor data sets with the same parameters used for the breast cancer data to get estimated false positive rates (Figure 6D). Filtered Strelka showed a surprising specificity (<0.01 false calls per million base pairs) though the sensitivity was limited. Virmid and MuTect also showed a satisfactory performance (approximately one per million base pairs). Unfiltered Strelka, which had almost comparable sensitivity for the breast cancer data as Virmid, however, produced more false positives (approximately three per million base pairs). JointSNVMix, even after applying its own filtering method, produced more false positives than the other tools.

We note that for the simulated data, Virmid's performance compared to the other tools was similar for samples with up to 50% contamination, but became progressively better for higher contamination levels such as those exceeding 70%. The experimental data presented here is at mid-levels of contamination (41% to 77%), which is not ideal for showing Virmid's strengths. In the next section, we discuss a new data set with higher levels of contamination, but without independently validated mutations. As the validated data sets grow in size, the advantages of calling mutations after estimating α are more apparent.

### Application to hemimegalencephaly exome sequencing data

We applied Virmid to the recently sequenced disease/normal paired data of five hemimegalencephaly (HME) patients [14]. HME is a rare disease characterized by the enlargement and malformation of one cerebral hemisphere and is known to be an important cause of epilepsy and developmental delay. One distinctive histopathological feature of HME is that dysmorphic and immature neurons are dispersed in a disease lesion. With this condition, brain samples from a surgical resection are expected to contain significant numbers of non-disease cells. Also, the mutation burden measured by whole exome sequencing and mass spectrometry from three previously validated mutations (AKT3 c.49C>T in HME-1565, MTOR c.4448C>T in HME-1563 and PIK3CA c.1633G>A in HME-1573) guarantee the compromised sample purity (see Table S1 in Additional file 1). The dropped allele frequencies (9.7% to 30.38%) are far less than expected (50% for heterozygous and 100% for homozygous mutation sites) indicating the existence of reference alleles (AA) from normal cells. As the mutations are believed to occur during early cerebral development and surgical treatment is carried out on infants for brain tissue acquisition, the low mutation burden is explained to a lesser degree by the disease subpopulation.

**Figure 7:**
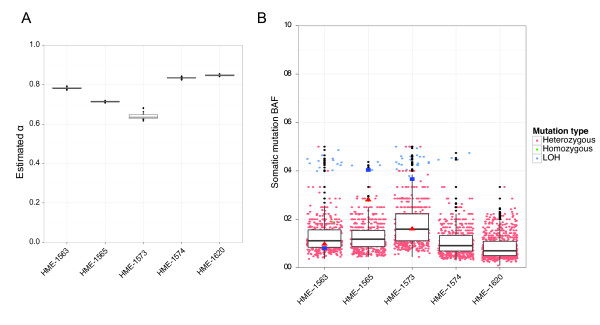
**Estimated α for HME samples**. Analysis of five hemimegalencephaly (HME) samples. **(A) **Estimated α values are shown as box plots. **(B) **BAF distributions of HME call sets. Each point represents one predicted somatic mutation in the corresponding sample. BAF was calculated from predicted heterozygous (orange) and homozygous (green) somatic mutations and loss of heterozygosity (LOH) sites. The mean BAF is consistent with the estimated α for every sample. The BAF calculated from read counts (red triangles) and mass spectrometry peak intensities (blue squares) of three previously validated mutations (MTOR, AKT3 and PI3KCA) are bounded in the predicted BAF ranges. BAF, B allele frequency; HME, hemimegalencephaly; LOH, loss of heterozygosity.

To estimate the sample contamination level, we ran Virmid 20 times for each whole exome sequencing data with various sampling parameters (see Materials and methods). The estimated α values for the five samples (HME-1563, HME-1565, HME-1573, HME-1574 and HME-1620) were surprisingly high, ranging from 64.0% to 84.8% (Figure 7A), which indicates that only 15.2% to 36% of each sample comprises diseased cells. The low variance (<0.075) within a sample gives a high confidence to the estimated values. While our manuscript was under review, an independent study [30] measured the mutation burden of this disease using 100 single-cell sequencing and reported a consistent result (39% in NeuN^+ ^and 27% in NeuN^- ^populations). We also checked the distribution of BAF at the site of candidate somatic mutations (Figure 7B). Note the overall drop of BAF towards zero as shown in the high α simulated examples (Figure 5 right). The average BAF was perfectly negatively correlated with the expected BAF calculated from (1 - α)/2. For example, the sample HME-1573, which was predicted to have the lowest contamination (α = 64.0%), had the highest BAF distribution. This negative correlation is consistent with our assumption that higher α leads to lower BAF. Although there is no efficient way to measure the true contamination levels in the sample, the allele frequencies of validated mutations (AKT3, MTOR and PIK3CA) provide a good reference. In HME-1563 and HME-1573, the validated BAF values (Figure 6B, red triangles) were very close to the expected heterozygous BAF. In HME-1565, the validated BAF (28%) is twice the expected BAF. We checked the genotype probability of the corresponding mutation and found that its probability of being homozygous was significantly high (7.1%, ranked second out of 496 mutations). In all cases, mutation burdens measured from mass spectrometry peak intensities (Figure 6B, blue squares) were also bounded in a low BAF range (8% to 40%). Although the allele frequencies at a single position were still too variable for a sample level estimator, we are convinced that the HME samples contain large numbers of normal cells and the estimated α values are reasonably ranged by aggregating measurements. Identifying and quantifying more somatic mutations will lead to better validation of the sample contamination level.

Finally, we ran Virmid on the same data set to find novel somatic mutations that might have been missed due to sample impurity. Virmid predicted a total of 2,787 somatic mutations from five individual data sets, only 653 (23.4%) of which were predicted previously [14] using JointSNVMix2 (see Table 3). Virmid called more novel (and less overlapping) mutations in samples with higher α (HME-1574 and HME-1620). Note that the number of newly predicted mutations is correlated with the estimated α; the higher α, the more somatic mutations could be missed from conventional approaches. Out of 2,134 newly predicted sites, 1,377 were located in exon regions containing 923 missense events. As expected, Virmid successfully discovered all the previously reported *de novo *mutations of PIK3CA, AKT3 and MTOR with very high confidence (*P *was approximately 1.0). We focused on the two (HME-1574 and HME-1620) samples where no meaningful somatic missense mutations were detected in the previous study. For HME-1574, Virmid discovered a novel somatic mutation of MTOR (MTOR p.Ala1517Thr) located near one of the validated mutations (MTOR p.Cys1483Tyr). At this site, only 3 out of 54 mapped reads represented B allele (BAF = 5.56%) while no B allele was found in the control sample; we could not find any sign of mapping ambiguity nor compromised base call quality. We are convinced that the extremely low BAF is the major reason for the unsuccessful results of conventional approaches. A functional analysis showed that the region is highly conserved (phastCons [31] score = 1.0) and no other mutation has been found at the same site so far. We expect further experimental validation will verify the mutation in other patients as well as determining more accurately the effects on protein activity (for example, gain of function). Virmid also detected more candidates for *de novo *somatic mutations in HME-1620, but we could not find meaningful missense mutations directly linked to the PI3K-ATK-mTOR pathway or HME pathogenesis. This is possibly either because disease-causing mutations exist other than in coding regions or because of the combinatorial effect of the low coverage of current exome sequencing (approximately 30 ×) and the high α (84.8%). As we showed in the simulation (Table 2), a much higher coverage (approximately 100 ×) might be necessary to ensure a predictive power to retrieve sufficient candidate mutations in such a highly impure sample.

**Table 3:** Newly predicted somatic mutations in hemimegalencephaly data.

Subject	Estimated α (standard deviation)	n.Total.Mut^a^	Overlapping mutation				Novel mutation			
			
			n.Mut^b^	n.Exon^c^	n.Mis^d^	Gene	n.Mut^b^	n.Exon^c^	n.Mis^d^	Gene
HME-1563	77.9% (±0.006)	478	108	54	33	MTOR	370	209	147	
HME-1565	71.3% (±0.003)	494	124	78	50	AKT3	370	72	43	
HME-1573	64.0% (±0.017)	542	235	167	112	PIK3CA	307	301	185	
HME-1574	83.4% (±0.005)	579	100	63	34		479	412	281	MTOR
HME-1620	84.8% (±0.004)	694	86	56	27		608	383	267	

^a^n.Total.Mut is the number of all called mutations.

^b^n.Mut is the number of (overlapping or novel) mutations. One mutation loci may be counted multiple times when more than one genetic product exists at the position.

^c^n.Exon is the number of mutations in an exon.

^d^n.Mis is the number of missense (or nonsense) mutations.

## Conclusions

In this study, we presented a novel probabilistic method for SNV calling, with two significant contributions. First, Virmid can estimate sample composition accurately or the level of contamination of a disease sample without genotyping. This not only saves time and money, but removes the severe estimation biases that come from initial SNV calling. Second, Virmid increases genotyping accuracy, especially somatic mutation profiling, by rigorously integrating the sample composition parameter into the SNV calling model. We showed that Virmid outperformed all recent tools in finding somatic mutations particularly in highly contaminated samples. In applying Virmid to HME disease/normal paired data sets, we discovered previously unknown sample impurity and somatic mutations. Our results suggest that it is important to estimate sample composition for all tumor/normal paired data when the sample purity is questionable and explicitly consider the composition in SNV calling if the sample is highly contaminated (>50%). The robustness of Virmid to high contamination also makes it applicable for identifying mutations in other challenging cases, such as with the low amounts of fetal DNA in maternal plasma.

## Materials and methods

### Virmid model

Virmid consists of three parts: the α estimator, G estimator and caller (Figure 1). In the α estimator and G estimator, we use the maximum likelihood estimation (MLE) method. The caller calls somatic variants using the Bayesian inference with the estimated joint genotype probability matrix as the prior distribution. To describe the estimation methods in detail, we first define the likelihood function and then describe how the likelihood function is used in each part of Virmid.

#### Likelihood function

Denote the set of the reads from the control sample by *C*. Given *C *and a nucleotide position *i*, the values of the reads mapped to the position *i *are represented by a vector (read vector) denoted by *C^i ^*where the *j*th element of *C^i ^*(*C^i^_j_*) is given by the value (that is, A or B) of the *j*th mapped read. For the set of the reads from disease-control mixture sample *D*, *D^i ^*is defined similarly.

For the likelihood function, the parameters are α and G. α is the proportion of control in the disease sample. The joint genotype probability matrix G is a 3 × 3 matrix that describes the average rate of the control-disease joint genotypes *g *(control genotype) and *g' *(disease genotype). Given a joint genotype *g *and *g'*, the element of G that corresponds to the joint genotype is specified by G*_g_*,*_g'_*. For example, G_AA,AB _is the rate at which the joint genotype of a random position is AA and AB. Note that G is not position-specific: it describes the average rate of each joint genotype over all positions. The position-specific rates are calculated in the caller using G as the prior distribution of joint genotypes (see below).

We assume that: (i) reads at different positions are independent and (ii) reads at the same position are independent given the joint genotype of that position. Given *θ *:= {α, G}, the likelihood function is written as:

(1) $$ℒ\left(\theta |C,D\right)=P_{\theta }\left(C,D\right)=\underset{i}{\prod }\left(\underset{\left(g,g^{\prime }\right)\in G}{\sum }\left\{\underset{\left(a\right)}{\underset{︸}{P_{\theta }\left(g,g^{\prime }\right)}}\cdot \overset{\left|C^{i}\right|}{\underset{j=1}{\prod }}\underset{\left(b\right)}{\underset{︸}{P_{\theta }\left(C_{j}^{i}|g\right)}}\cdot \overset{\left|D^{i}\right|}{\underset{j=1}{\prod }}\underset{\left(c\right)}{\underset{︸}{P_{\theta }\left(D_{j}^{i}|g,g^{\prime }\right)}}\right\}\right)$$

where *G *is the set of all possible joint genotypes, and |*C^i^*| and |*D^i^*| denote the read depths of *C^i ^*and *D^i^*, respectively (see the supplementary note in Additional file 1 for the derivation of the likelihood function).

The probability given by expression (*a*) of Equation (1) is defined by *P_θ_*(*g*,*g*'):= G*_g_*,*_g_*'. The probabilities in (*b*) and (*c*) are defined so that their definitions incorporate the read error rate, mapping error rate and the LOR bias. The LOR bias is that reads with more mismatches are less mappable to the reference sequence, which is due to the maximum number for the allowed edit distance. First we derive the probabilities in (*b*) and (*c*) without considering the LOR bias. Denote the read and mapping error probability of *C^i^_j _*by *r *and *m*, respectively.

The probability *P_θ_*(*C^i^_j_*|*g*) is defined by:

(2) $$P_{\theta }\left(C_{j}^{i}|g\right):=\left\{\begin{array}{c} m\left(\frac{1}{4}\cdot r+\left(1-r\right)\beta \right)+\left(1-m\right)\left(\frac{1}{4}\cdot r+\left(1-r\right)\gamma \right)\;\;\;\;\;\;\;\;\text{if}\;C_{j}^{i}=\text{A}\\ 1-m\left(\frac{1}{4}\cdot r+\left(1-r\right)\beta \right)-\left(1-m\right)\left(\frac{1}{4}\cdot r+\left(1-r\right)\gamma \right)\;\text{if}\;C_{j}^{i}=\text{B} \end{array}\right)$$

where *γ *is the probability that an error-free read (that is, without mapping or read errors) has the A allele given *g *and *β *is the probability that an incorrectly mapped read has the A allele. *γ *is calculated as:

(3) $$\gamma =\left\{\begin{array}{c} 1\;\;\;\;\;\;\;\;\;\;\;\;\;\;\;\;\;\;\;\text{if}\;g=\text{AA}\\ \frac{1}{2}\;\;\;\;\;\;\;\;\;\;\;\;\;\;\;\;\;\text{if}\;g=\text{AB}\\ 0\;\;\;\;\;\;\;\;\;\;\;\;\;\;\;\;\;\;\text{if}\;g=\text{BB}\text{.} \end{array}\right)$$

*β *may depend on experimental settings but is simply set to be *β *= 0.99. For simplicity, we denote the right-hand side of Equation (2) as the function *μ_g_*(*C^i^_j_*).

The probability *P_θ_*(*D^i^_j_*|*g*,*g*') in (*c*) is given by:

(4) $$P_{\theta }\left(D_{j}^{i}|g,g^{\prime }\right):=\alpha \mu_{g}\left(D_{j}^{i}\right)+\left(1-\alpha \right)\mu_{g^{\prime }}\left(D_{j}^{i}\right).$$

The right-hand side of Equation (4) is denoted by *v_g_^g'^*(*D^i^_j_*, α).

Second we derive the probabilities in (*b*) and (*c*) considering the LOR bias. Denote the probability that a read with at least one A (or B) allele is mappable (that is, the edit distance of the read is less than the maximum allowed edit distance) by *x*(A) (or *x*(B)). Then we obtain:

(5) $$P_{\theta }\left(C_{j}^{i}|g\right)=\frac{\mu_{g}\left(C_{j}^{i}\right)\cdot x\left(C_{j}^{i}\right)}{\mu_{g}\left(\text{A}\right)\cdot x\left(\text{A}\right)+\mu_{g}\left(\text{B}\right)\cdot x\left(\text{B}\right)}$$

The right-hand side of Equation (5) is denoted by *f_g_*(*C^i^_j_*). We also obtain:

(6) $$P_{\theta }\left(D_{j}^{i}|g,g^{\prime }\right)=\frac{v_{g}^{g^{\prime }}\left(D_{j}^{i},\alpha \right)\cdot x\left(D_{j}^{i}\right)}{v_{g}^{g^{\prime }}\left(\text{A,}\alpha \right)\cdot x\left(\text{A}\right)+v_{g}^{g^{\prime }}\left(\text{B,}\alpha \right)\cdot x\left(\text{B}\right)}$$

The right-hand side of Equation (6) is denoted by *h_g_^g'^*(*D^i^_j_*, α). A detailed derivation of the above probabilities, *x*(*C^i^_j_*), *μ_g_*(*C^i^_j_*), *v_g_^g'^*(*D^i^_j_*, α), *f_g_*(*C^i^_j_*), and *h_g_^g'^*(*D^i^_j_*, α), is given in the supplementary note in Additional file 1.

Overall, we have:

(7) $$ℒ\left(\theta |C,D\right)=\underset{i}{\prod }\left(\underset{\left(g,g^{\prime }\right)\in G}{\sum }\left\{G_{g,g^{\prime }}\cdot \overset{\left|C^{i}\right|}{\underset{j=1}{\prod }}f_{g}\left(C_{j}^{i}\right)\cdot \overset{\left|D^{i}\right|}{\underset{j=1}{\prod }}h_{g}^{g^{\prime }}\left(D_{j}^{i},\alpha \right)\right\}\right).$$

#### Basic model

Using the likelihood function defined above, the MLE of *θ *= (α, G) can be obtained from:

(8) $$\hat{\theta }=\arg\;\max\;ℒ\left(\theta |C,D\right)$$

with the right constraints (for example, 0 ≤ α ≤ 1 and G_AA,AA _> 100 G_AA,AB _- see the supplementary note in Additional file 1 for the constraints used). Since the exact global maximum point cannot be derived analytically, one needs to use a numerical approach to find it. To make a numerical approach work, one should carefully avoid local maximum points. However, even if we impose strong constraints, many local maximum points may be present in the likelihood function. Moreover, in terms of the estimation of α, not all read vectors are useful: some read vectors deteriorate the estimation (see below). Therefore, we try to estimate α and then estimate all elements in G (with the estimated α).

#### Estimation of α

For the estimation of α, the disease read vectors generated from the same control genotype (*g*) and disease genotype (*g*') are simply noisy sample points conveying no information. Thus, we want to sample the read vectors generated from different *g *and *g*', but without the initial calling. Also we want to fix *g *=AA so that the number of parameters to be estimated can be minimized. Denote the number of B's in a control read vector *C^i ^*(or in a disease read vector *D^i^*) by <*C^i^*> (or <*D^i^*>). We sample the positions *i *such that <*C^i^*> = 0 and <*D^i^*>/|*D^i^*| > R (that is, the BAF of *D^i ^*is larger than *R*) for a real value 0 <*R *≤ 1. Imposing <*C^i^*> = 0 minimizes the chance that *g *=AA, and imposing <*D^i^*>/|*D^i^*| > R increases the chance that *g*' =AA.

If *R *is too large, however, we may not have sufficient samples for the estimation. On the other hand, if *R *is too small, the samples may contain too many read vectors from *g *= AA and *g*' = AA that serve as noise. Thus, we estimate α using different values of *R *and take the median of the estimates. Table 1 shows that our α estimator is quite robust for different values of *R*. We also output the asymptotic variance of the estimated α using the outer products of the first de-rivatives of the log likelihoods (which is called the BHHH estimator [32]).

With the selected samples as above, we only estimate four parameters (instead of 10 - α and 9 elements in G): α, G_AA,AA_, G_AA,AB _and G_AA,BB_; other elements in G are set to a very small number close to 0. In this step, the parameters except α are estimated simply to give a better estimation of α.

Unfortunately, the sampling described above introduces estimation bias if we use the likelihood function as is because the sampling procedure inflates the number of B's in the disease read vector (see Figure S2 in Additional file 1 for an example). To take this sampling bias into account, we modify the likelihood function as:

(9) $${ℒ}_{R}\left(\theta |C,D\right)=P_{\theta }\left(C,D|\frac{\left\langle D^{i}\right\rangle }{\left|D^{i}\right|}>R\;\text{for}\;\text{all}\;i\right)=\underset{i}{\prod }\left(\underset{\left(g,g^{\prime }\right)\in G}{\sum }\left\{G_{g,g^{\prime }}\cdot \overset{\left|C^{i}\right|}{\underset{j=1}{\prod }}f_{g}\left(C_{j}^{i}\right)\cdot \underset{\left(d\right)}{\underset{︸}{\frac{\overset{\left|D^{i}\right|}{\underset{j=1}{\prod }}h_{g}^{g^{\prime }}\left(D_{j}^{i},\alpha \right)}{P_{\theta }\left(\frac{\left\langle D^{i}\right\rangle }{\left|D^{i}\right|}>R|g,g^{\prime }\right)}}}\right\}\right).$$

The denominator in (*d*) can be efficiently calculated using a dynamic programming algorithm with time complexity of O(*R *· |*D^i^*|^2^) (see the supplementary note in Additional file 1 for the algorithm). As above, the parameter *R *can be readily incorporated in our model (and used to correct the possible bias); however, it is very hard to produce a rigorous model that takes the LOV bias found in the calling-based methods into account.

The estimates of the four parameters ($\hat{\alpha }$,  Ĝ_AA,AA_,  Ĝ_AA,AB _and  Ĝ_AA,BB_) that maximize the likelihood are found by the feasible direction method [33], a gradient descent search method with constraints. Note that only the estimate of α is retained for the next step.

Figure 3A shows the values of the log likelihood over different $\hat{\alpha }$ and  Ĝ_AA,AB _(for each point, other parameters are optimized). For low α, the optimum  Ĝ_AA,AB _is almost 1. However, when α is larger, the likelihood is maximized for low  Ĝ_AA,AB_. For example, when α = 0.9, the maximum likelihood is found when  Ĝ_AA,AB _≈ 0.1. Such estimation results are predicted because for high α, even disease read vectors generated with *g*' = AB would not have a sufficient number of B's to distinguish between *g*' = AB and *g' *= AA. Even if we sample disease read vectors with many B's, there are often many vectors from *g*' =AA, which leads to a high value of  Ĝ_AA,AB_.

#### Estimation of the joint genotype probability matrix

In this step, we estimate G with the estimated α. We sample 1,000,000 positions except ones at which the number of B's in the disease read vector is zero (that is, <*D^i^*> = 0). Such positions are not sampled because Virmid does not analyze such points for SNP calling. We estimate  Ĝ to maximize the likelihood function in (7) using the feasible direction method.

#### Calling genotypes

Given the estimated $\hat{\theta }=\left(\hat{\alpha },\;\;Ĝ\right)$ and a position *i*, we first calculate G*^i^*, the posterior distribution of genotypes at the position *i *(with  Ĝ as the prior distribution), by:

(10) $$G_{g,g^{\prime }}^{i}=P_{\hat{\theta }}\left(g,g^{\prime }|C^{i},D^{i}\right)$$

(11) $$=\frac{P_{\hat{\theta }}\left(C^{i},D^{i}|g,g^{\prime }\right)\cdot P_{\hat{\theta }}\left(g,g^{\prime }\right)}{\underset{\left(k,k^{\prime }\right)\in G}{\sum }P_{\hat{\theta }}\left(C^{i},D^{i}|k,k^{\prime }\right)\cdot P_{\hat{\theta }}\left(k,k^{\prime }\right)}$$

(12) $$=\frac{{\hat{G}}_{g,g^{\prime }}\cdot \overset{\left|C^{i}\right|}{\underset{j=1}{\prod }}f_{g}\left(C_{j}^{i}\right)\cdot \overset{\left|D^{i}\right|}{\underset{j=1}{\prod }}h_{g}^{g^{\prime }}\left(D_{j}^{i},\hat{\alpha }\right)}{\underset{\left(k,k^{\prime }\right)\in G}{\sum }{\hat{G}}_{k,k^{\prime }}\cdot \overset{\left|C^{i}\right|}{\underset{j=1}{\prod }}f_{k}\left(C_{j}^{i}\right)\cdot \overset{\left|D^{i}\right|}{\underset{j=1}{\prod }}h_{k}^{k^{\prime }}\left(D_{j}^{i},\hat{\alpha }\right)}.$$

Then Virmid calls the position *i *a somatic variant if

$$1-\left(G_{\text{AA},\text{AA}}^{i}+G_{\text{AB},\text{AB}}^{i}+G_{\text{BB},\text{BB}}^{i}\right)>0.5$$

#### Filtration of read data

Reads or positions that may contain unreliable information were filtered out from the observation. Two filtering criteria have been established and they are used in different Virmid steps. The first filtering scheme is used when selecting observation points for α estimation. The purpose of filtering in this step is to eliminate positions that possibly contain the following types of noise: (1) B alleles originating from a sequencing error, (2) B alleles originating from a mapping error and (3) B alleles originating from the non-reference control genotype. The second filtering scheme is used for calling somatic mutations. In this step, it removes false-positive somatic mutations, which are usually due to one of the following cases: (1) both samples have the reference genotype (AA-AA) but B alleles are observed through sequencing or mapping errors and (2) both samples have a non-reference genotype (AB-AB, germ line mutation) but significant BAF differences are observed. To satisfy these conditions, mutations for all frequent miscalling events are filtered out based on seven criteria:

1. Mapping quality (MQ): If the mapping quality of the corresponding B allele read is significantly worse (>30 MAPQ score) than for A allele reads or if the overall ratio of ambiguously mapped reads (<17 MAPQ score) is above a threshold (>0.4).

2. Read offset filter: If the position of a B allele in a read is significantly biased at the both ends (z-score > 3).

3. Indel proximity (PRX): If more than 50% of the B alleles are located within 10 bp of nearby indels.

4. Tri-allele (TRI): If the major allele frequency is less than 0.9.

5. Base quality (BQ): If the mean base call quality of a B allele read is less than 20.

6. Number of mismatches (NM): If the mean number of mismatches per read is more than 3 or if more than 60% of the reads are soft or hard clipped.

7. Allele frequency (AF): If the absolute number of B alleles is less than a threshold (3) or BAF in the control is more than one tenth (1/10) of that in the disease sample.

The filters are applied differently in α estimation and mutation calling. For α estimation, our goal is to eliminate germ line mutations (AB-AB) by only allowing reference and somatic mutation alleles. To do so, we strictly apply the MQ, PRX and NM filters to prevent potential mapping errors. For mutation calling, we apply all seven filters with empirically known parameters. These parameters can be also defined by a user.

### Data preparation

#### Simulated data

First, two diploid genomes were simulated: a normal genome and a disease genome. The normal genome was created by using the hg19 genome as a template and infusing germ line SNPs found in dbSNP 135 [34] at a rate of one SNP per thousand nucleotides. Somatic mutations were introduced by perturbing a nucleotide to any of the other three nucleotides with equal probability at rates of 10^-5 ^mutations per nucleotide to simulate a disease genome. Both of these simulations were carried out using in-built Python functions. The Python scripts are available upon request.

GemSim v1.5 [35] was set up to generate paired-end 100 bp reads using the Illumina paired-end error model. The number of reads required was calculated using the average coverage of the sample (40 × and 100 ×). The metagenomic mode was configured with four genomes: normal haplotype 1, normal haplotype 2, disease haplotype 1 and disease haplotype 2. The relative abundance of each genome was calculated based on the contamination level (α = 1%, 5%, 10%, 20%, 30%, 40%, 50%, 60%, 70%, 80% and 90%). For the normal sample, the metagenomic mode was configured with normal haplotype 1 and normal haplotype 2 in equal abundances. All the reads were aligned using bwa [36] and passed through the GATK data-processing pipeline for variant calling including indel realignment and base quality score recalibration. The resulting BAM files were fed into the variant calling tools.

#### Breast cancer data

There were 545 tumor to normal matched samples with verified somatic mutations. The putative somatic mutations were validated using the Illumina Capture gDNA technologies. To assess normal cell contamination in tumor specimens, we downloaded the matched tumor/normal samples listed as whole exome sequencing (WXS) on CGHub [37] under controlled access. Since essential post-processing on the mapping, such as indel realignment and quality recalibration, is time consuming, we limited our analysis to 15 randomly selected patients as our gold standard (see Table S2 in Additional file 1 for a full list). The 1,654 validated mutations were extracted from the accompanying mutation annotation format (MAF) file using the following criteria: (1) field 'Validation Status' (column 25) is 'Valid' and (2) field 'Variant Type' (column 10) is 'SNP'.

#### HME data

Five paired normal data sets (10 BAM files, 76 bp, 30 × coverage) processed in the previous study [14] were downloaded with the authors' permission. The alignments had already been post-processed using GATK's pipeline including IndelRealigner, MarkDuplicate and TableRecalibration. Pileup files were generated using SAMtools mpileup and indexed with tabix. Possible noise reads that did not pass the quality check or were possibly included as PCR duplicates were filtered out using the -F view option in SAMtools.

#### Call-based estimation of α

Initial SNV calling was done using Virmid without α. All the filtration steps were applied after the initial calling. The detailed calculation steps are given in the supporting information of Snyder *et al*. [15]. Briefly, the number of reads at the called somatic mutation sites are classified by genotype and allele type. The donor fraction is estimated from:

$$\frac{2N_{\text{AB}}\left(\text{B}\right)+N_{\text{BB}}\left(\text{B}\right)}{N_{\text{AB}}\left(\text{A}\right)+N_{\text{AB}}\left(\text{B}\right)+N_{\text{BB}}\left(\text{A}\right)+N_{\text{BB}}\left(\text{B}\right)}$$

where *N_G_*(*A*) denotes the number of reads at the site of genotype *G *with the A allele.

#### Somatic mutation call sets

Strelka version 0.4.5 was used for the comparative studies. The program was configured using the provided settings for bwa. The results presented show the calls after the first filtration step and after the final filtration step. For JointSNVMix version 0.7 [8], the results were generated using the JointSnvMix2 mode, which accounts for base and mapping errors. First the program was trained using the jsm.py *train *option, and then joint genotype calls were made using the jsm.py *classify *option. All of the configurable settings were left with their default values. For the Area Under the Curve (AUC), we varied the probability cutoff necessary to make a joint genotype call to adjust the specificity and sensitivity of the program. For the filtered version of JointSNVMix2, we used JointSNVMix version 0.8 with the '-post process option'. Other parameters were the same as JointSNVMix version 0.7. We evaluated the performance of VarScan2 [7] using version 2.2.11. The pileup files were created using SAMtools version 0.1.18 [2]. The somatic option was adjusted with the Virmid-derived values of contamination for tumor purity calculations. Note that VarScan2 takes a parameter for sample purity but only as a pre-calculated form. In this case, we used Virmid's estimation (α) to feed it. We also carried out additional filtering using the default options for VarScan2's methods *somaticFilter *and *processFilter *as well as the Perl script fpfilter.pl, which is available on VarScan2's website. The most recent version of bam-readcount from the GitHub repository was used to create the input files for the Perl script. Lastly, we ran MuTect as described in the MuTect website, except for the '-cosmic' option since the validated mutations (true answer) were included in the corresponding database.

### Program implementation and optimization

We implemented the Virmid model and its surrounding workflow using Java (JDK version 1.6), SAMtools and the Picard library. Post-processed BAM files were converted to pileup format using SAMtools' mpileup program. Mapping quality scores were included in the pileup files using the '-s' option. To optimize the overall pipeline, we divided the pileup data into three different layers (Figure S1A in Additional file 1). Pileup level 1 is the most fundamental data where the B allele was observed at least once in the disease genome. Level 2 data contains all the positions where the observed BAF is higher than or equal to 5% as well when no B allele was observed in the control genome. Lastly, level 3 data was generated by increasing the minimum BAF until the number of satisfying positions was less than a threshold (generally 1,000 to 10,000). α was estimated using the level 3 disease pileup data. After getting α, we called genotypes of the positions included in the level 1 pileup files. This hierarchical model significantly reduced the overall search space (Figure S1B in Additional file 1). Starting from all the nucleotide regions of chromosome 1 (approximately 240 Mbp), the level 1 data was reduced to 9% of its original size. The final number of data points was 0.28% and 0.00041% of the original number in the level 2 and level 3 data, respectively. Due to the successful reduction, we decreased the running time for α estimation to less than a few minutes.

## List of abbreviations used

AF: allele frequency; BAF: B allele frequency; bp: base pair; BQ base quality; CNV: copy number variation; GATK: Genome Analysis Toolkit; HME: hemimegalencephaly; LOH: loss of heterozygosity; LOR: loss of reads; LOV: loss or variants; MLE: maximum likelihood estimator; MQ: mapping quality; NM: number of mismatches; PRX: indel proximity; SNP: single nucleotide polymorphism; SNV: single nucleotide variation; TCGA: The Cancer Genome Atlas; TRI: tri-allele.

## Competing interests

The authors declare that they have no competing interests.

## Authors' contributions

SK, KJ, KB, VB designed the base model. SK and KJ implemented the model. SK and KB generated the simulated data and tested the tool. SK, KJ and VB prepared the manuscript. JL and JGG motivated the problem and reviewed the biological discoveries. AP prepared and processed the breast cancer data set. ES worked on the functional analysis of the discovered mutations. HN worked on data analysis and presentation. HL wrote a module for genome mappability score. All authors read and approved the final manuscript.

## Supplementary Material

Additional file 1 **Supplementary information**. A supplementary Portable Document File (PDF) that includes supplementary figures (Figures S1 to S4), tables (Tables S1 and S2) and methods (supplementary notes 1 to 5 including mathematical proofs, derivations and model descriptions). Click here for file
