# Supplementary material for: Virmid: accurate detection of somatic mutations with sample impurity inference
Source: Genome Biol. 2013 Aug 29;14(8):R90. doi: 10.1186/gb-2013-14-8-r90 (PMC4054681; doi:10.1186/gb-2013-14-8-r90)
Supplement: Additional file 1 — Supplementary information. A supplementary Portable Document File (PDF) that includes supplementary figures (Figures S1 to S4), tables (Tables S1 and S2) and methods (supplementary notes 1 to 5 including mathematical proofs, derivations and model descriptions). [file gb-2013-14-8-r90-S1.PDF]

Supplementary Information for "Virmid: accurate detection of somatic mutations with sample impurity inference"

Sangwoo Kim[1,7*], Kyowon Jeong[2,7] , Kunal Bhutani[1], Jeong Ho Lee[3,6], Anand Patel[1], Eric Scott[3], Hojung Nam[4], Hayan Lee[5], Joseph G Gleeson[3] and Vineet Bafna[1*]

[1]Department of Computer Science and Engineering, University of California at San Diego, 9500 Gilman Drive, La Jolla, CA 92093, USA
[2]Department of Electrical and Computer Engineering, University of California at San Diego, 9500 Gilman Drive, La Jolla, CA 92093, USA
[3]Institute for Genomic Medicine, Rady Children's Hospital, University of California at San Diego, 9500 Gilman Drive, La Jolla, CA 92093, USA
[4]School of Information and Communications, Gwangju Institute of Science and Technology, 123 Cheomdangwagi-ro, Buk-gu, Gwangju, 500-712, Republic of Korea
[5]Department of Computer Science, Stony Brook University, 100 Nicolls Road, NY 11794, USA
[6]Graduate School of Medical Science and Engineering, KAIST, 291 Daehak-ro, Yuseong-gu, Daejeon 305-701, Republic of Korea
[7]These authors contributed equally to this work

Email: Sangwoo Kim - sak042@cs.ucsd.edu; Kyowon Jeong - kwj@ucsd.edu; Kunal Bhutani - kbhutani@eng.ucsd.edu; Jeong Ho Lee - jhlee4246@kaist.ac.kr; Anand Patel - adp002@ucsd.edu; Eric Scott - escott55@gmail.com; Hojung Nam - hjnam@gist.ac.kr; Hayan Lee - hlee@cshl.edu; Joseph G Gleeson - jogleeson@ucsd.edu; Vineet Bafna - vbafna@cs.ucsd.edu;

*Corresponding author

Contents

1 Supplementary Figures **2**

2 Supplementary Tables **6**

3 Supplementary notes **8**

3.1 Derivation of the likelihood function . . . 8

3.2 Derivation of the probability $P_\theta(C_j^i|g)$ . . . 8

3.3 Derivation of the probability $P_\theta(D_j^i|g, g')$ . . . 10

3.4 Constraints for the joint genotype probability matrix $\mathcal{G}$ . . . 11

3.5 Dynamic programming algorithm to calculate $P_\theta\left(\frac{\langle D^i \rangle}{|D^i|} > R|g, g'\right)$ . . . 12

List of Figures

S1 Multi-tier sampling of Virmid . . . 2

S2 Sampling bias with BAF filter and correction . . . 3

S3 Analog of Figure 3 for different α's . . . 4

S4 Analog of Figure 3 for different α's (Cont'd) . . . 5

List of Tables

S1 Mutation burden of validated somatic mutations in HME . . . 6

S2 A list of 15 public breast cancer data from TCGA. . . . 7

1 Supplementary Figures

Figure S1: Multi-tier sampling of Virmid. A, given disease BAM file is first reduced to a smaller subset in which at least one B allele is observed. In control BAM sample, only positions in which no B allele is observed are used. These samples are further filtered out using minimum BAF to increase the probability of selecting true somatic mutations. B, the size of sample is dramatically reduced down to 0.01% of the initial data.

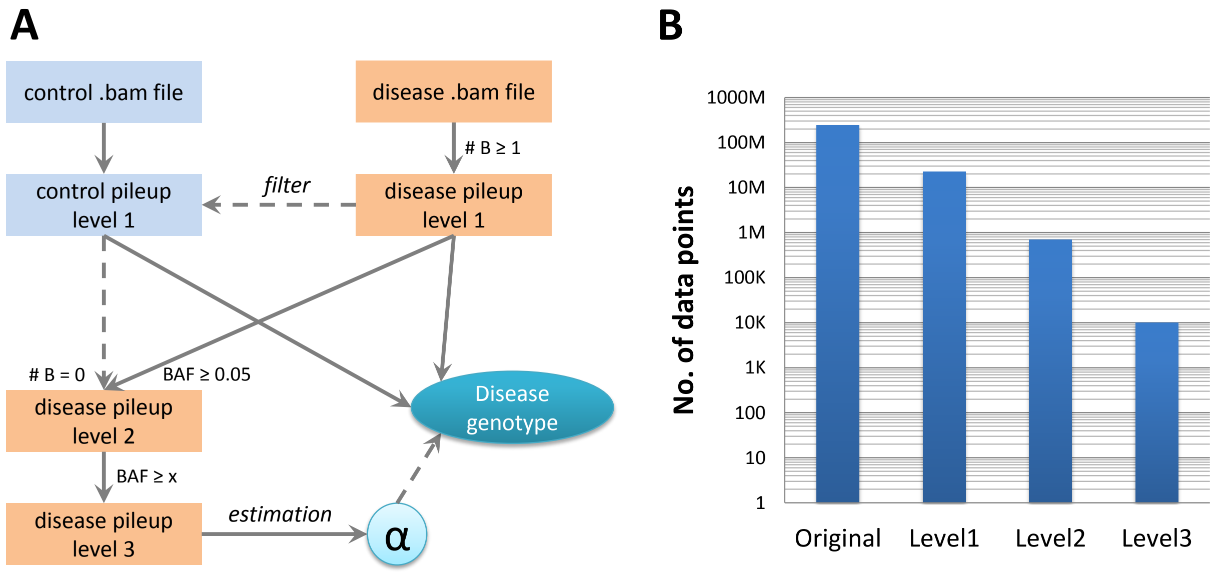

Figure S2: An example of the sampling bias of α estimation in a tumor reads of depth 20. Suppose that the disease read vector is generated with joint genotype $g =$AA and $g' =$BB and that α is 0.9. Then, ignoring errors, the number of A alleles in the read vector can be modeled by the binomial distribution $B(20, 0.9)$ (blue bars). Thus, if we simply estimate α by the portion of A alleles in this read vector, the estimate is expected to be 0.9 (unbiased). However, if this read vector passed the sampling using $R = 0.2$, the number of A alleless is always less than or equal to 16. The probability that the number of A alleless is k is given by by $\frac{B(20,0.9)(k)}{\sum_{j\leq 16} B(20,0.9)(j)}$ if $k \leq 16$ and by 0 otherwise (red bars). Using this probability mass function, the estimated α (i.e., the expected portion of A alleles) is obtained as 0.74, which is significantly lower than 0.9.

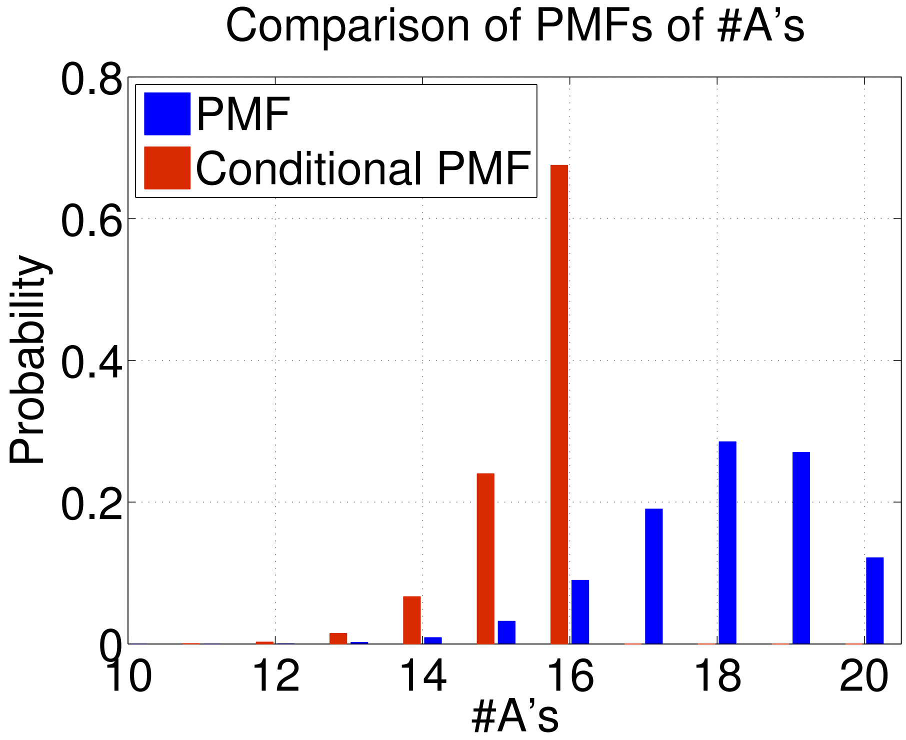

Figure S3: Analog of Figure 3 for different α's

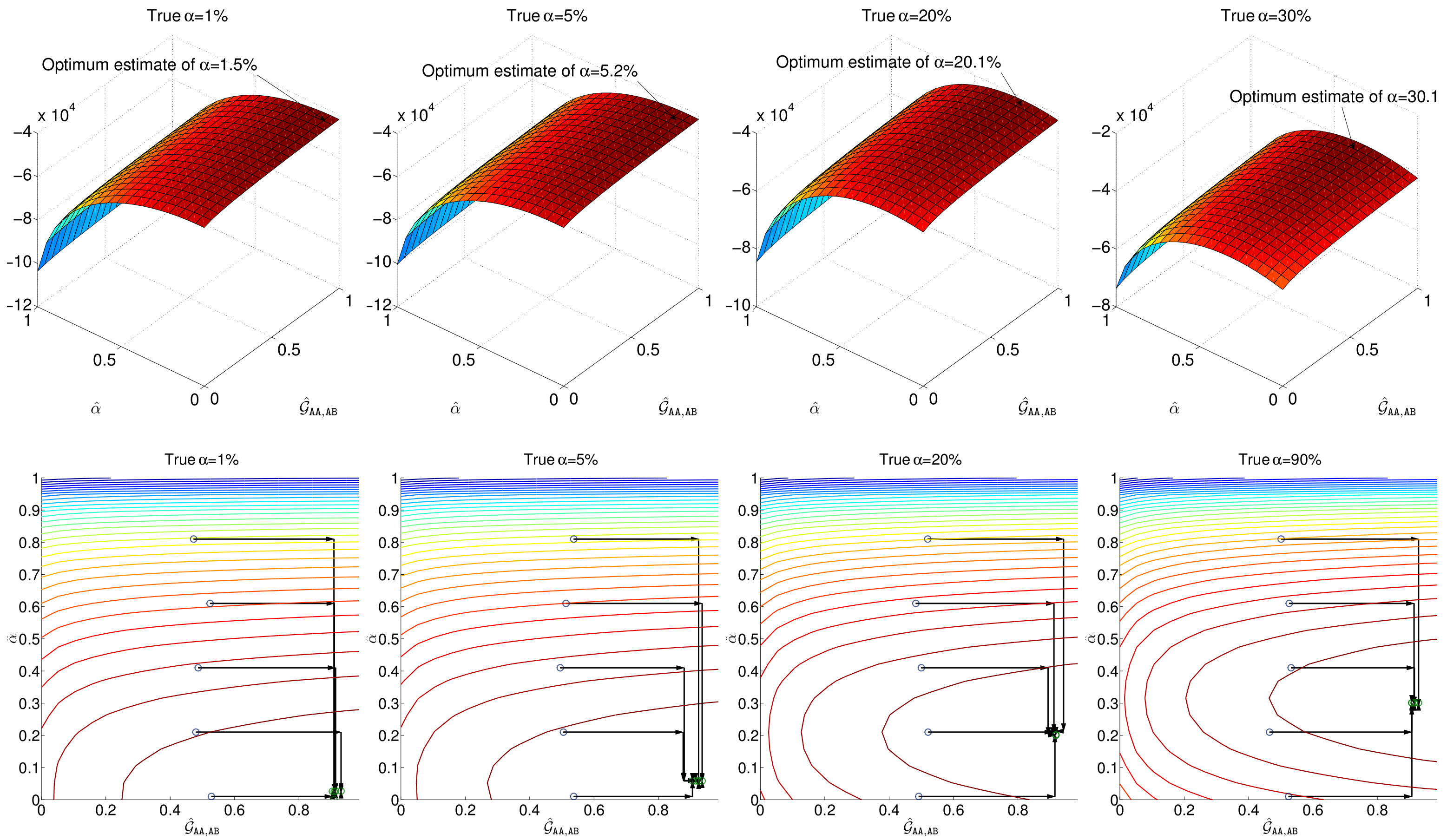

Figure S4: continued

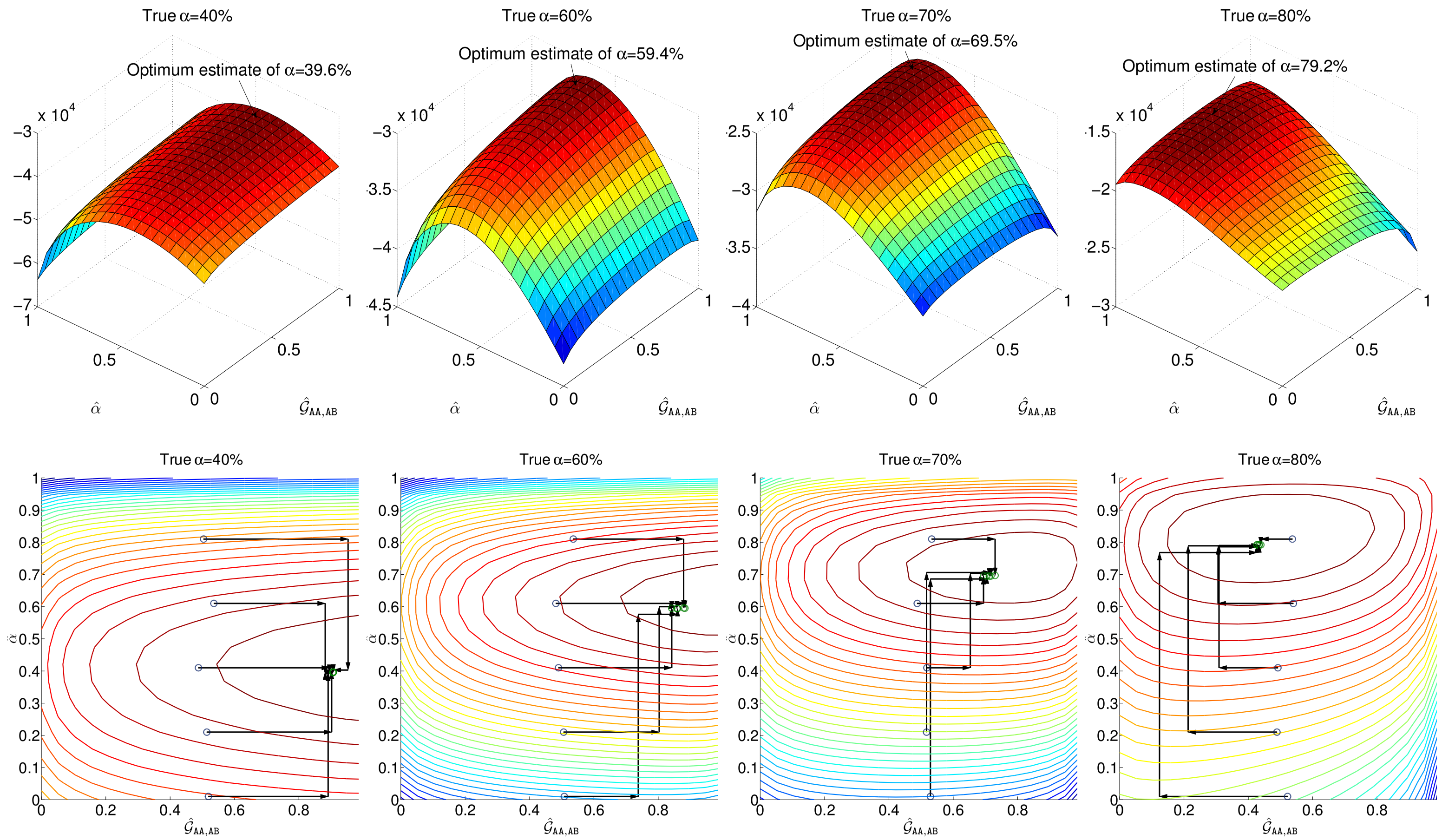

2 Supplementary Tables

Table S1: Three previously validated mutations have deviated mutation burdens compared to the expected values (50%). The mutation burdens were measured using mass spectrometry (MS) or whole exome sequencing (WES) from previous study. Samples are collected from different sites of the affected brain hemisphere. CO=Central operculum, Pa=Parietal, Te=Temporal, Or=Orbital, Fr=Frontal and Oc=Occipital

Subject	Mutation	Method	Mutation burden (Sampling lesion)	Average (%)
HME-1563	MTOR c.4448C>T	MS	30.3%, 8.3%, 36.4%, 9.1%, 18.1% (CO)	20.44%
		WES	9.7% (17/176)	9.7%
HME-1565	AKT3 c.49C>T	MS	26.9% (Pa), 30.4% (CO), 15.6% (Te)	24.3%
		WES	28.0% (9/32)	28.0%
HME-1573	PIK3CA c.1633G>A	MS	25.0% (Or), 36.0% (Fr), 39.5% (CO), 21.0% (Oc)	30.38%
		WES	16.0% (9/56)	16.0%

Table S2: A list of 15 public breast cancer data from TCGA.

Tumor sample ID	Normal sample ID (matched)
TCGA-BH-A0DZ-01A-11W-A019-09	TCGA-BH-A0DZ-10A-01W-A021-09
TCGA-A2-A0T0-01A-22D-A099-09	TCGA-A2-A0T0-10A-01D-A099-09
TCGA-A1-A0SO-01A-22D-A099-09	TCGA-A1-A0SO-10A-03D-A099-09
TCGA-A8-A06X-01A-21W-A019-09	TCGA-A8-A06X-10A-01W-A021-09
TCGA-A8-A093-01A-11W-A019-09	TCGA-A8-A093-10A-01W-A021-09
TCGA-AO-A0J9-01A-11W-A050-09	TCGA-AO-A0J9-10A-01W-A055-09
TCGA-AN-A0FJ-01A-11W-A019-09	TCGA-AN-A0FJ-10A-01W-A021-09
TCGA-A8-A095-01A-11W-A019-09	TCGA-A8-A095-10A-01W-A021-09
TCGA-BH-A0DK-01A-21W-A071-09	TCGA-BH-A0DK-10A-01W-A071-09
TCGA-A7-A0CE-01A-11W-A019-09	TCGA-A7-A0CE-10A-01W-A021-09
TCGA-AR-A0TV-01A-21D-A099-09	TCGA-AR-A0TV-10A-01D-A099-09
TCGA-A8-A0A7-01A-11W-A019-09	TCGA-A8-A0A7-10A-01W-A021-09
TCGA-BH-A0C0-01A-21W-A071-09	TCGA-BH-A0C0-10A-01W-A071-09
TCGA-AO-A0JL-01A-11W-A071-09	TCGA-AO-A0JL-10A-01W-A071-09
TCGA-A2-A0CT-01A-31W-A071-09	TCGA-A2-A0CT-10A-01W-A071-09
TCGA-A8-A07L-01A-11W-A019-09	TCGA-A8-A07L-10A-01W-A021-09

3 Supplementary notes

3.1 Derivation of the likelihood function

We assume i) all reads at different positions are independent and ii) all reads at the same position are independent given the joint genotype of that position.

The likelihood function is given by

$$
\begin{aligned}
\mathcal{L}(\theta|C,D) &= P_\theta(C,D) && (1)\\
&= \prod_i P_\theta(C^i,D^i) && (2)\\
&= \prod_i \Big(\sum_{(g,g')\in G} P_\theta(C^i,D^i,g,g') \Big) && (3)\\
&= \prod_i \Big(\sum_{(g,g')\in G} P_\theta(g,g')\cdot P_\theta(C^i,D^i|g,g') \Big) && (4)\\
&= \prod_i \Big(\sum_{(g,g')\in G} P_\theta(g,g')\cdot P_\theta(C^i|g)P_\theta(D^i|g,g') \Big) && (5)\\
&= \prod_i \Big(\sum_{(g,g')\in G} \Big\{ \underbrace{P_\theta(g,g')}_{(a)} \cdot \prod_{j=1}^{|C^i|} \underbrace{P_\theta(C^i_j|g)}_{(b)} \cdot \prod_{j=1}^{|D^i|} \underbrace{P_\theta(D^i_j|g,g')}_{(c)} \Big\} \Big). && (6)
\end{aligned}
$$

The equations between (1) and (2), (4) and (5), and (5) and (6) are from the above independence assumptions.

3.2 Derivation of the probability $P_\theta(C^i_j|g)$

Before we derive the probability $P_\theta(C^i_j|g)$ we first define $x(\texttt{A})$ (or $x(\texttt{B})$), the probability that a read with at least one A (or B) allele is mappable (i.e., the edit distance of the read is less than the maximum allowed edit distance). Denote the number of mismatches in a read with read length l by #B. The distribution of #B given one A or B allele in the read can be derived regorously per read using the error rates; however, for simplicty, we assume it follows $B(l-1,p)$, the Binomial distribution with $l-1$ trials and the success probability of p. p is the average probability of observing mismatches. We set $p = 0.008$.

Given a maximum number of allowed edit distance d, $x(\texttt{A})$ is given by

$$
\begin{aligned}
x(\texttt{A}) &= P_\theta(\#\texttt{B} \le d|\text{an A allele is observed})\\
&= \sum_{j=0}^{d} \binom{l-1}{j} p^j (1-p)^{l-1-j}.
\end{aligned}
$$

Likewise, $x(\texttt{B})$ is given by

$$x(\texttt{A}) = \sum_{j=0}^{d-1} \binom{l-1}{j} p^j (1-p)^{l-1-j}.$$

To take this probability $x(\cdot)$ into account for $P_\theta(C_j^i|g)$, we think that the read vector (at position i) is generated by two steps: i) generation of a *raw* read vector and ii) generation of the read vector from the raw read vector. The raw read vector is the same as the read vector except that $x(\texttt{A})$ is assumed to equal $x(\texttt{B})$ (i.e., the LOR bias is ignored). Then in the second step each element in a raw read vector is retained in the read vector with probability $x(\texttt{B})$ when the element corresponds to a mismatch or with $x(\texttt{A})$ otherwise. Thus, for each element in the read vector C_j^i, there is a corresponding element (that C_j^i is from) in the raw read vector. Denote the corresponding element of C_j^i by $\hat{C}_j^i$.

We first derive $P_\theta(\hat{C}_j^i|g)$. Suppose $\hat{C}_j^i = \texttt{A}$. Define two Bernoulli random variables E_r and E_m as

$$E_r = \begin{cases} 1 & \text{if a read error has occurred for } \hat{C}_j^i \\ 0 & \text{otherwise} \end{cases}$$

and

$$E_m = \begin{cases} 1 & \text{if a mapping error has occurred for } \hat{C}_j^i \\ 0 & \text{otherwise.} \end{cases}$$

We have $P_\theta(E_r = 1) = r$ and $P_\theta(E_r = 1) = m$. The parameter β (the probability that an incorrectly mapped read has Aallele) can be written by $P_\theta(\hat{C}_j^i = \texttt{A}|E_m = 1, E_r = 0)$, and γ (the probability that an error-free read has A allele given g) by $P_\theta(\hat{C}_j^i = \texttt{A}|E_m = 0, E_r = 0, g)$. β is simply defined by 0.99, and γ is by

$$\gamma = \begin{cases} 1 & \text{if } g = \texttt{AA} \\ \frac{1}{2} & \text{if } g = \texttt{AB} \\ 0 & \text{if } g = \texttt{BB}. \end{cases}$$

It is assumed that $\hat{C}_j^i$ and the genotype g are independent when $E_r = 1$ or $E_m = 1$.

Now for $\hat{C}^i_j$ =A, we have

$$P_\theta(\hat{C}^i_j = \mathtt{A}|g) \tag{7}$$

$$= P_\theta(\hat{C}^i_j = \mathtt{A}, E_m = 1|g) + P_\theta(\hat{C}^i_j = \mathtt{A}, E_m = 0|g) \tag{8}$$

$$= P_\theta(\hat{C}^i_j = \mathtt{A}, E_m = 1) + P_\theta(\hat{C}^i_j = \mathtt{A}, E_m = 0|g) \tag{9}$$

$$= P_\theta(E_m = 1) \cdot P_\theta(\hat{C}^i_j = \mathtt{A}|E_m = 1) + P_\theta(E_m = 0|g) \cdot P_\theta(\hat{C}^i_j = \mathtt{A}|E_m = 0, g) \tag{10}$$

$$= m \cdot P_\theta(\hat{C}^i_j = \mathtt{A}|E_m = 1) + (1-m) \cdot P_\theta(\hat{C}^i_j = \mathtt{A}|E_m = 0, g) \tag{11}$$

$$= m \cdot (r \cdot P_\theta(\hat{C}^i_j = \mathtt{A}|E_m = 1, E_r = 1) + (1-r) \cdot P_\theta(\hat{C}^i_j = \mathtt{A}|E_m = 1, E_r = 0)) + (1-m) \cdot P_\theta(\hat{C}^i_j = \mathtt{A}|E_m = 0, g) \tag{12}$$

$$= m \cdot (\frac{1}{4} \cdot r + (1-r)\beta) + (1-m) \cdot P_\theta(\hat{C}^i_j = \mathtt{A}|E_m = 0, g) \tag{13}$$

$$= m \cdot (\frac{1}{4} \cdot r + (1-r)\beta) + (1-m) \cdot (r \cdot P_\theta(\hat{C}^i_j = \mathtt{A}|E_m = 0, E_r = 1, g) + (1-r) \cdot P_\theta(\hat{C}^i_j = \mathtt{A}|E_m = 0, E_r = 0, g)) \tag{14}$$

$$= m \cdot (\frac{1}{4} \cdot r + (1-r)\beta) + (1-m) \cdot (\frac{1}{4} \cdot r + (1-r)\gamma). \tag{15}$$

If $\hat{C}^i_j$ =B,

$$P_\theta(\hat{C}^i_j = \mathtt{B}|g) = 1 - \big(m \cdot (\frac{1}{4} \cdot r + (1-r)\beta) + (1-m) \cdot (\frac{1}{4} \cdot r + (1-r)\gamma)\big). \tag{16}$$

To derive $P_\theta(C^i_j|g)$ from $P_\theta(\hat{C}^i_j|g)$, we define a Bernoulli random variable I_m such that

$$I_m = \begin{cases} 1 & \text{if } \hat{C}^i_j \text{ is retained in the read vector} \\ 0 & \text{otherwise .} \end{cases}$$

We have $P_\theta(I_m = 1|\hat{C}^i_j = \mathtt{A}) = x(\mathtt{A})$ and $P_\theta(I_m = 1|\hat{C}^i_j = \mathtt{B}) = x(\mathtt{B})$. Since C^i_j we observe is always retained from $\hat{C}^i_j$ (i.e., $I_m = 1$ is given), the probability $P_\theta(C^i_j|g)$ can be rewritten by $P_\theta(\hat{C}^i_j|g, I_m = 1)$, which is proportional to $P_\theta(\hat{C}^i_j|g) \cdot P_\theta(I_m = 1|\hat{C}^i_j)$. Therefore, if we denote $P_\theta(\hat{C}^i_j|g)$ by $\mu_g(\hat{C}^i_j)$, we obtain

$$P_\theta(C^i_j|g) = \frac{\mu_g(C^i_j) \cdot x(C^i_j)}{\mu_g(\mathtt{A}) \cdot x(\mathtt{A}) + \mu_g(\mathtt{B}) \cdot x(\mathtt{B})} \tag{17}$$

This probability is denoted by $f_g(C^i_j)$.

3.3 Derivation of the probability $P_\theta(D^i_j|g, g')$

Denote the corresponding element of D^i_j in the raw read vector by $\hat{D}^i_j$ as above. Define a Bernoulli random variable I_C by

$$I_C = \begin{cases} 1 & \text{if } \hat{D}^i_j \text{ is from the control sample} \\ 0 & \text{otherwise .} \end{cases}$$

The success probability of I_C is given by α. We have

$$P_\theta(\hat{D_j^i}|g,g') = P_\theta(\hat{D_j^i}, I_C = 0|g,g') + P_\theta(\hat{D_j^i}, I_C = 1|g,g') \tag{18}$$

$$= P_\theta(\hat{D_j^i}, I_C = 0|g') + P_\theta(\hat{D_j^i}, I_C = 1|g) \tag{19}$$

$$= (1-\alpha)P_\theta(\hat{D_j^i}|I_C = 0, g') + \alpha P_\theta(\hat{D_j^i}|I_C = 1, g) \tag{20}$$

$$= (1-\alpha)\mu_{g'}(\hat{D_j^i}) + \alpha\mu_g(\hat{D_j^i}). \tag{21}$$

Denote $P_\theta(\hat{D_j^i}|g,g')$ by $\nu_g^{g'}(\hat{D_j^i},\alpha)$. As in (17), we obtain

$$P_\theta(D_j^i|g,g') = \frac{\nu_g^{g'}(D_j^i,\alpha)\cdot x(D_j^i)}{\nu_g^{g'}(\mathtt{A},\alpha)\cdot x(\mathtt{A}) + \nu_g^{g'}(\mathtt{B},\alpha)\cdot x(\mathtt{B})} \tag{22}$$

This probability is denoted by $h_g^{g'}(D_j^i,\alpha)$.

3.4 Constraints for the joint genotype probability matrix $\mathcal{G}$

We use several linear constraints on the parameters α and $\mathcal{G}$. The constraints are different in the α estimation step and $\mathcal{G}$ estimation step. This is because in the α estimation step we are only estimating α and a subset of $\mathcal{G}$. Moreover, some of elements of $\mathcal{G}$ are expected to have different frequencies in the α estimation step than in the $\mathcal{G}$ estimation step. We remark that the constraints we used are relatively liberal; they were used just to reduce the search space.

Constraints for α estimation

$$0 \leq \alpha \leq 1$$

$$0 \leq \mathcal{G}_{\mathtt{AA},\mathtt{AA}} \leq 1$$

$$0 \leq \mathcal{G}_{\mathtt{AA},\mathtt{AB}} \leq 1$$

$$0 \leq \mathcal{G}_{\mathtt{AA},\mathtt{BB}} \leq 1$$

$$10 \cdot \mathcal{G}_{\mathtt{AA},\mathtt{BB}} \leq \mathcal{G}_{\mathtt{AA},\mathtt{AB}}$$

$$\mathcal{G}_{\mathtt{AA},\mathtt{AA}} + \mathcal{G}_{\mathtt{AA},\mathtt{AB}} + \mathcal{G}_{\mathtt{AA},\mathtt{BB}} = 1$$

Constraints for $\mathcal{G}$ estimation

$$
\begin{aligned}
0 \leq & \alpha \leq 1 \\
0 \leq & \mathcal{G}_{g,g'} \leq 1 \text{ for } g, g' \in \{\texttt{AA},\texttt{AB},\texttt{BB}\} \\
10^2 \cdot (\mathcal{G}_{\texttt{AB},\texttt{AA}} + \mathcal{G}_{\texttt{AB},\texttt{AB}} + & \mathcal{G}_{\texttt{AB},\texttt{BB}}) \leq (\mathcal{G}_{\texttt{AA},\texttt{AA}} + \mathcal{G}_{\texttt{AA},\texttt{AB}} + \mathcal{G}_{\texttt{AA},\texttt{BB}}) \\
10^2 \cdot (\mathcal{G}_{\texttt{BB},\texttt{AA}} + \mathcal{G}_{\texttt{BB},\texttt{AB}} + & \mathcal{G}_{\texttt{BB},\texttt{BB}}) \leq (\mathcal{G}_{\texttt{AB},\texttt{AA}} + \mathcal{G}_{\texttt{AB},\texttt{AB}} + \mathcal{G}_{\texttt{AB},\texttt{BB}}) \\
10^4 \cdot & \mathcal{G}_{\texttt{AA},\texttt{AB}} \leq \mathcal{G}_{\texttt{AA},\texttt{AA}} \\
10^2 \cdot & \mathcal{G}_{\texttt{AA},\texttt{BB}} \leq \mathcal{G}_{\texttt{AA},\texttt{AB}} \\
10^6 \cdot & \mathcal{G}_{\texttt{AB},\texttt{AA}} \leq \mathcal{G}_{\texttt{AB},\texttt{AB}} \\
10^2 \cdot & \mathcal{G}_{\texttt{AB},\texttt{BB}} \leq \mathcal{G}_{\texttt{AB},\texttt{AB}} \\
10^6 \cdot & \mathcal{G}_{\texttt{BB},\texttt{AB}} \leq \mathcal{G}_{\texttt{BB},\texttt{BB}} \\
10^6 \cdot & \mathcal{G}_{\texttt{BB},\texttt{AA}} \leq \mathcal{G}_{\texttt{BB},\texttt{BB}} \\
\sum_{g,g' \in \{\texttt{AA},\texttt{AB},\texttt{BB}\}} & \mathcal{G}_{g,g'} = 1
\end{aligned}
$$

3.5 Dynamic programming algorithm to calculate $P_\theta\Big(\frac{\langle D^i \rangle}{|D^i|} > R|g,g'\Big)$

Denote $R \cdot |D^i|$ by N. Then, $P_\theta\Big(\frac{\langle D^i \rangle}{|D^i|} > R|g,g'\Big) = P_\theta\Big(\langle D^i \rangle > N|g,g'\Big) = 1 - P_\theta\Big(\langle D^i \rangle \leq N|g,g'\Big)$. We calculate $P_\theta\Big(\langle D^i \rangle \leq N|g,g'\Big)$ using a dynamic programming.

Let $H(j,n)$ be the probability that the n elements among $D^i_1, \cdots, D^i_j$ are B alleles. Then, when $j > 1$ and $n > 0$ we have

$$H(j,n) = H(j-1,n) \cdot P_\theta(D^i_j = \texttt{A}|g,g') + H(j-1,n-1) \cdot P_\theta(D^i_j = \texttt{B}|g,g'). \tag{23}$$

Since $H(j,0) = H(j-1,0) \cdot P_\theta(D^i_j = \texttt{A}|g,g')$ for $n = 0$ and $P_\theta(D^i_j|g,g') = h^{g'}_g(D^i_j)$, we obtain the following recursion:

$$H(j,n) = H(j-1,n) \cdot h^{g'}_g(D^i_j = \texttt{A}, \alpha) + H(j-1,n-1) \cdot h^{g'}_g(D^i_j = \texttt{B}, \alpha) \tag{24}$$

for $n \geq 0$ and $j \geq 1$. The boundary conditions are given by $H(0,0) := 1$ and $H(j,-1) := 0$. The probability $P_\theta\Big(\frac{\langle D^i \rangle}{|D^i|} > R|g,g'\Big) = 1 - P_\theta\Big(\langle D^i \rangle \leq N|g,g'\Big)$ is calculated by $1 - \sum_{n=0}^{N} H(|D^i|,n)$. The time complexity is given by $O(|D^i| * N) = O(|D^i|^2 \cdot R)$.
